# Supplementary material for: Ultrasound-mediated delivery of doxorubicin to the brain results in immune modulation and improved responses to PD-1 blockade in gliomas
Source: Nat Commun. 2024 Jun 6;15:4698. doi: 10.1038/s41467-024-48326-w (PMC11156895; doi:10.1038/s41467-024-48326-w)
Supplement: Supplementary file 1 — Supplementary Information [file 41467_2024_48326_MOESM1_ESM.pdf]

Supplementary Table 1. Clinical characteristics of the GBM patient cohort included in the expanded access protocol (doxorubicin plus pembrolizumab)

IDH status	*MGMT*	Expanded Access Protocol	DOXIL Induction	DOX dose	Pembrolizumab dose	Number of cycles prior to surgery/biopsy
Wild type	Unmethylated	Doxil/ Keytruda (Pembrolizumab)	Yes	30 mg	200 mg	2 cycles
Wild type	Unmethylated	Doxil/ Keytruda (Pembrolizumab)	Yes	30 mg	200 mg	8 cycles
Wild type	Unmethylated	Doxil/ Keytruda (Pembrolizumab)	Yes	30 mg	200 mg	3 cycles
Wild type	Unmethylated	Doxil/ Keytruda (Pembrolizumab)	Yes	30 mg	200 mg	6 cycles

Supplementary Figure 1

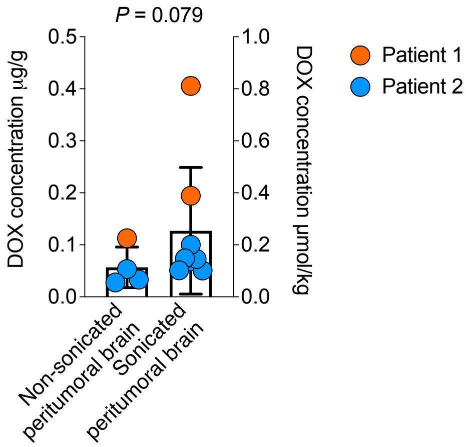

Supplementary Figure 1. LIPU/MB increases DOX concentrations in the human brain. a, Bar plot showing the concentrations of DOX in sonicated (n = 4 brain samples) and non-sonicated peritumoral brain regions (n = 8 brain samples) obtained during surgery after 48 hours of DOX infusion from 2 GBM patients. A mixed effects model was constructed considering sonication as a fixed effect and patients as a random effect influencing the fold change in DOX concentration. *P* value was obtained by a chi-squared test of the likelihood ratio test of the full model with sonication as the fixed effect against the model without the fixed effect. Source data are provided as a Source Data file. Data are presented as mean ± SEM.

Supplementary Figure 2

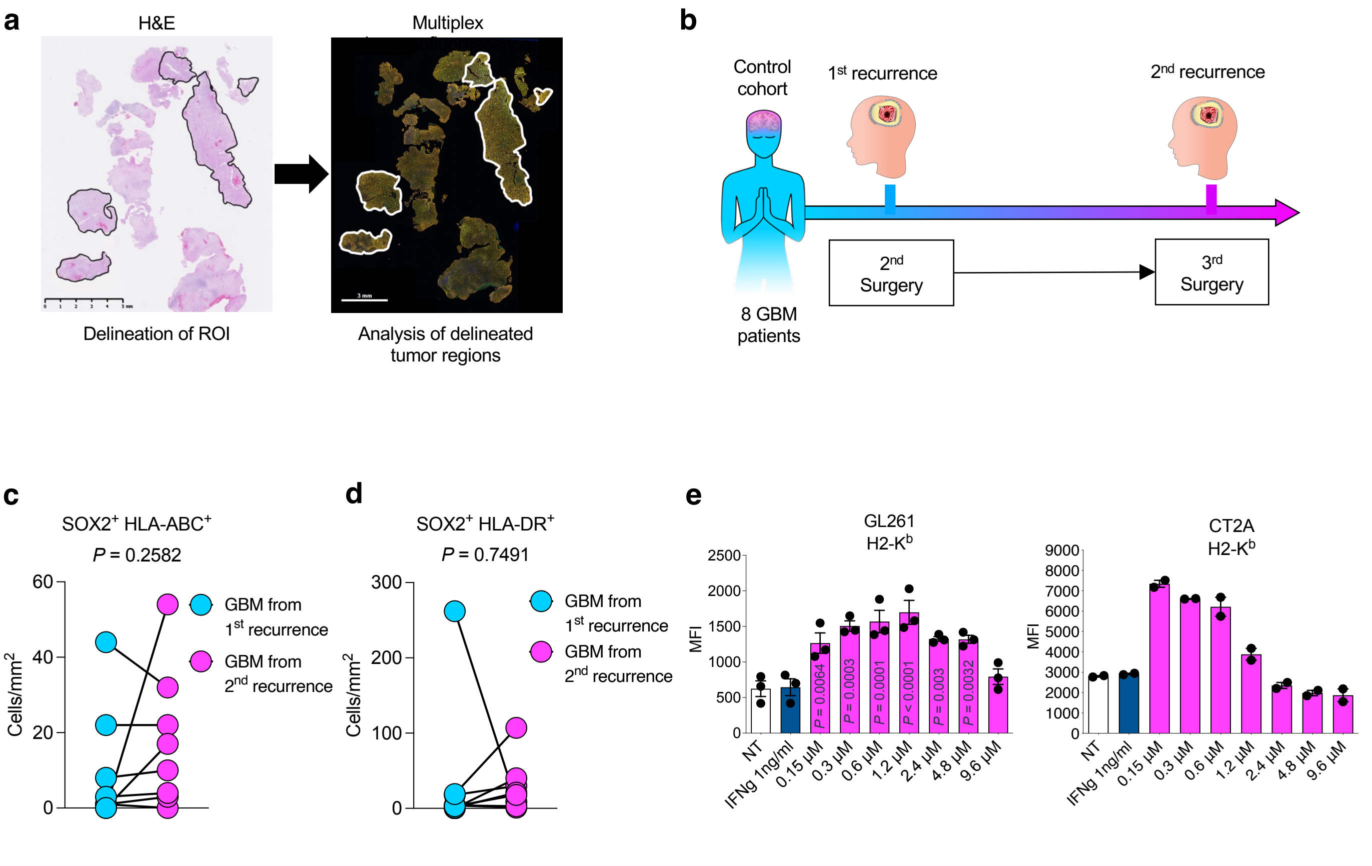

Supplementary Figure 2. DOX upregulates antigen-presenting molecules in GBM. a, Microscopy images of H&E staining and multiplex immunofluorescence staining representing tumor regions delineated by the neuropathologist used to quantify tumor and immune cell populations. **b**, Schematic representation of the patient cohort and the surgical timeline. Eight GBM patients underwent surgery at initial recurrence and second recurrence. **c**, **d**, Quantitative analysis of $SOX2^+$ $HLA\text{-}ABC^+$ (**c**) and $SOX2^+$ $HLA\text{-}DR^+$ (**d**) cell densities in GBM samples from the first and second recurrences. A mixed effects model was constructed considering recurrence as a fixed effect and patients as a random effect influencing cell densities from the indicated phenotypes. *P* values were obtained by chi-squared tests of likelihood ratio tests of the full model with the fixed effect against the model without the fixed effect. **e**, Bar plots representing the expression of $H2\text{-}K^b$ assessed as MFI values in GL261 (n = 3 biological replicates per group) and CT-2A (n = 2 biological replicates per group). Error bars represent standard deviation, and P-values indicate the statistical significance of the treated groups compared to the control group derived from one-way ANOVA with post hoc Dunnett's multiple comparisons test in **e.** Source data are provided as a Source Data file.

Supplementary Figure 3

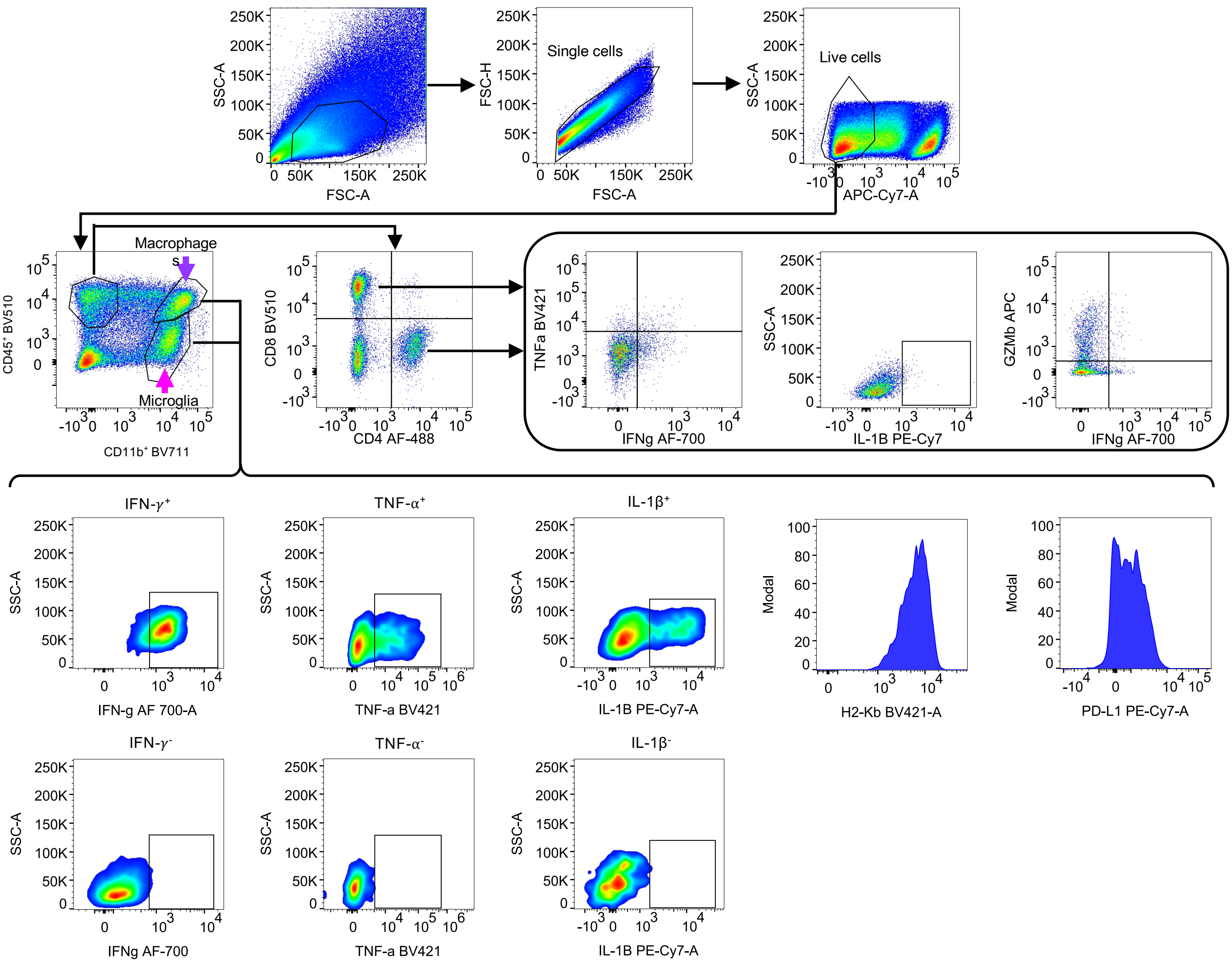

Supplementary Figure 3. Gating strategy used to analyze blood and GBM-infiltrating immune cells in mice treated with different doses of liposomal DOX with and without LIPU/MB. Flow cytometry plots used to analyze the production of cytokines and the expression of surface markers in murine immune cells. Immune cells were gated based on SSC and FSC parameters followed by the exclusion of doublets. Next, live cells were gated based on low values of APC-Cy7 fluorescence intensity. Subsequently, live cells were gated based on the expression of CD45$^+$ and CD11b$^+$. Then, lymphocytes were gated based on CD45$^+$ and CD11b$^-$ followed by gating employing CD8$^+$ and CD4$^+$ markers. IFN-γ^+, GZMb$^+$, TNF-α^+ and IL-1β^+ were evaluated on CD8$^+$ and CD4$^+$ T cells. Macrophages were gated based on CD45$^+$ and CD11b$^+$. Microglia were gated based on CD45^{+dim} and CD11b$^+$. Macrophages and microglia were interrogated for the production IFN-γ^+, TNF-$\alpha^{+,}$ and IL-1β^+ as well as the expression of H2-Kb$^+$ and PD-L1$^+$.

Supplementary Figure 4

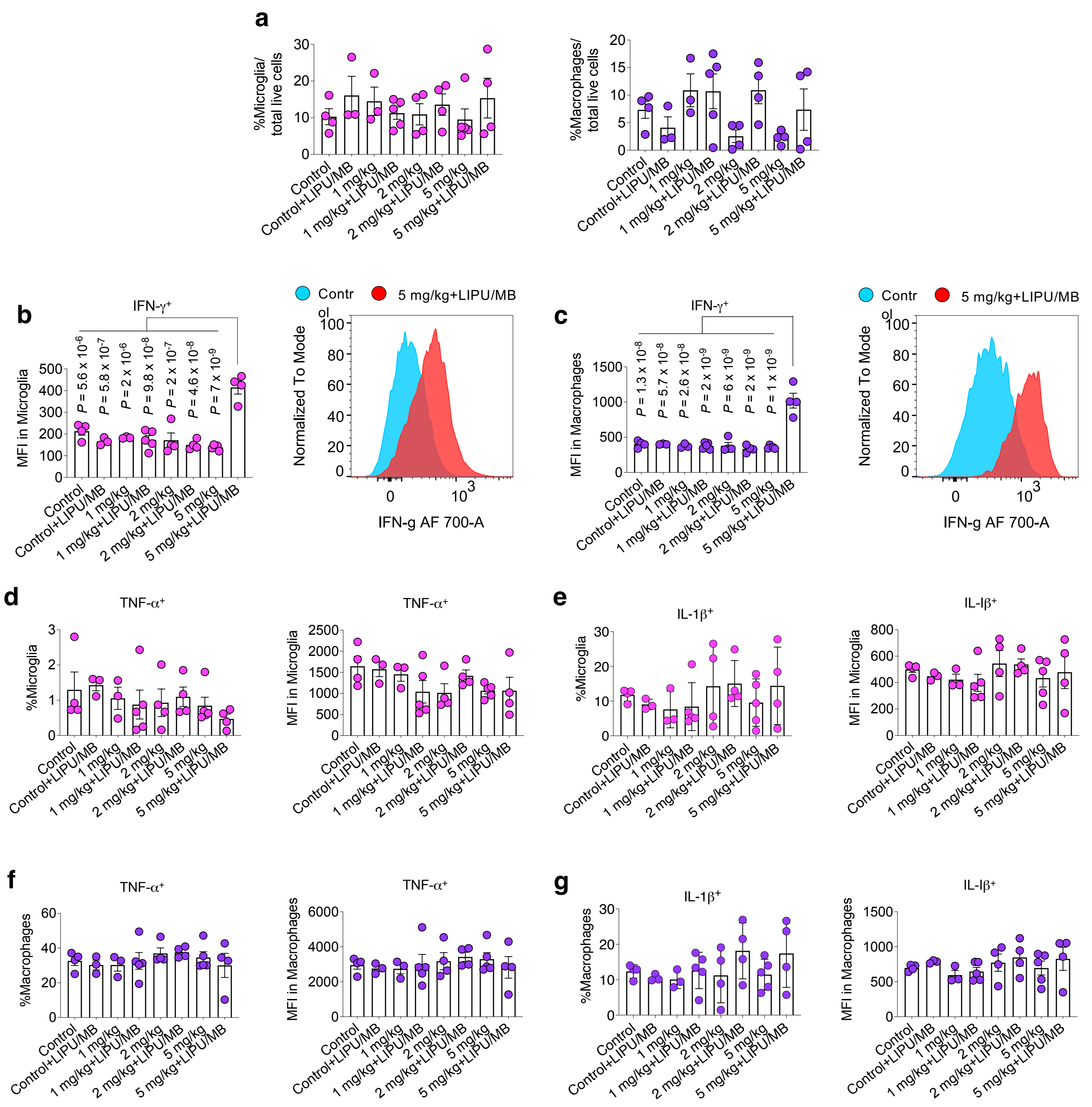

Supplementary Figure 4. Effect of liposomal DOX delivered with and without LIPU/MB in tumor-infiltrating immune cells. a, Bar plots showing the percentages of microglia (left) and macrophages (right) from groups treated with different doses of liposomal DOX (1, 2, and 5 mg/kg) with or without LIPU/MB. **b**, **c**, Bar plots and histograms showing the MFI of IFN-γ+ from groups treated with different doses of liposomal DOX (1, 2, and 5 mg/kg) with or without LIPU/MB in microglia (left) and macrophages (right). **d, e,** Bar plots showing the percentage of cells TNF-α+ (**d**) and IL-1β+ (**e**) from groups treated with different doses of liposomal DOX (1, 2, and 5 mg/kg) with or without LIPU/MB in microglia. **f, g,** Bar plots showing the percentage of cells TNF-α+ (**f**) and IL-1β+ (**g**) from groups treated with different doses of liposomal DOX (1, 2, and 5 mg/kg) with or without LIPU/MB in macrophages. n=3 mice for control+LIPU/MB and 1mg/kg; n=4 mice for control, 2 mg/kg, 2 mg/kg+LIPU/MB, and 5 mg/kg+LIPU/MB; and n=5 mice for 1 mg/kg+LIPU/MB and 5 mg/kg. All samples were derived from biologically independent mice from 1 experiment. Source data are provided as a Source Data file. Data are presented as mean ± SEM in **a**, **b**, **c**, **d**, **e**, **f**, and **g**. *P* values in **a**, **b**, **c**, **d**, **e**, **f**, and **g** were derived from one way-ANOVA with post hoc Tukey's multiple comparisons test.

Supplementary Figure 5

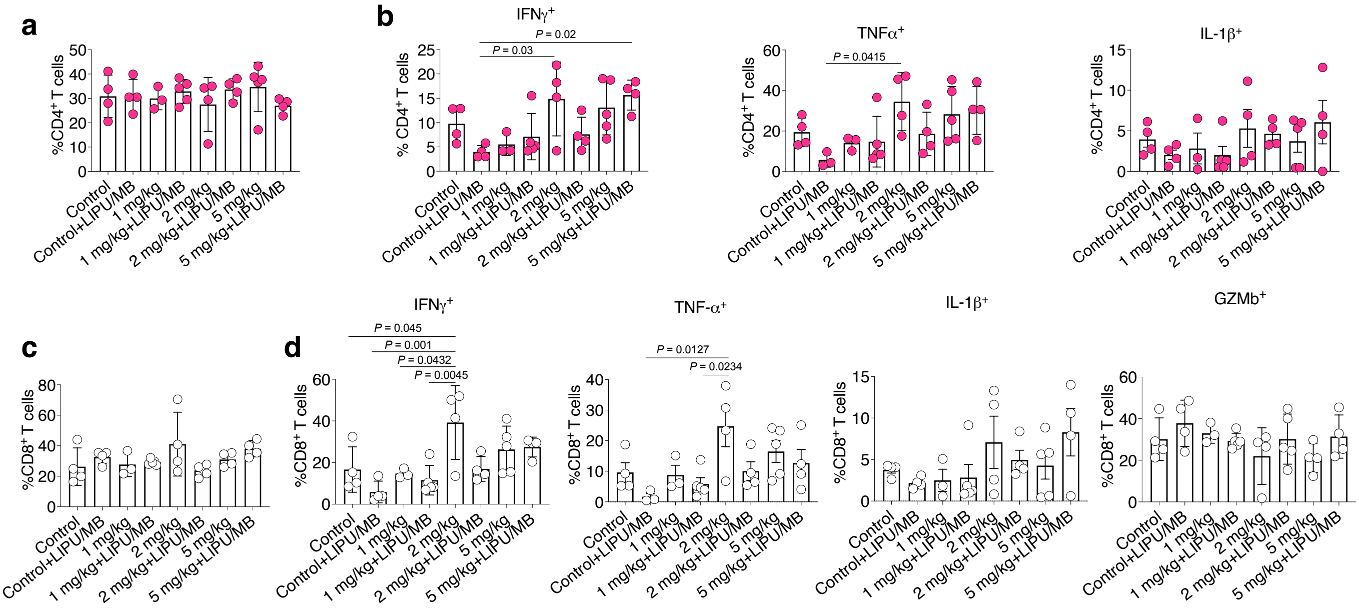

Supplementary Figure 5. Effect of liposomal DOX in tumor-infiltrating T cells. a, b, Bar plots representing the percentage of CD4+ (**a**) and those that express IFN-γ+, TNF-α+, and IL-1β+ (**b**) from groups treated with different doses of liposomal DOX (1, 2, and 5 mg/kg) with or without LIPU/MB. **c, d,** Bar plots representing the percentage of CD8+ (**c**) T cells, and those that express IFN-γ+, TNF-α+, IL-1β+ and GZMb (**d**) from groups treated with different doses of liposomal DOX (1, 2, and 5 mg/kg) with or without LIPU/MB. n=3 mice for control+LIPU/MB and 1mg/kg; n=4 mice for control, 2 mg/kg, 2 mg/kg+LIPU/MB, and 5 mg/kg+LIPU/MB; and n=5 mice for 1 mg/kg+LIPU/MB and 5 mg/kg. All samples were derived from biologically independent mice from 1 experiment. Source data are provided as a Source Data file. Data are presented as mean ± SEM in **a**, **b**, **c,** and **d**. *P* values in **a**, **b**, **c**, and **d** were derived from one way-ANOVA with post hoc Tukey's multiple comparisons test.

Supplementary Figure 6

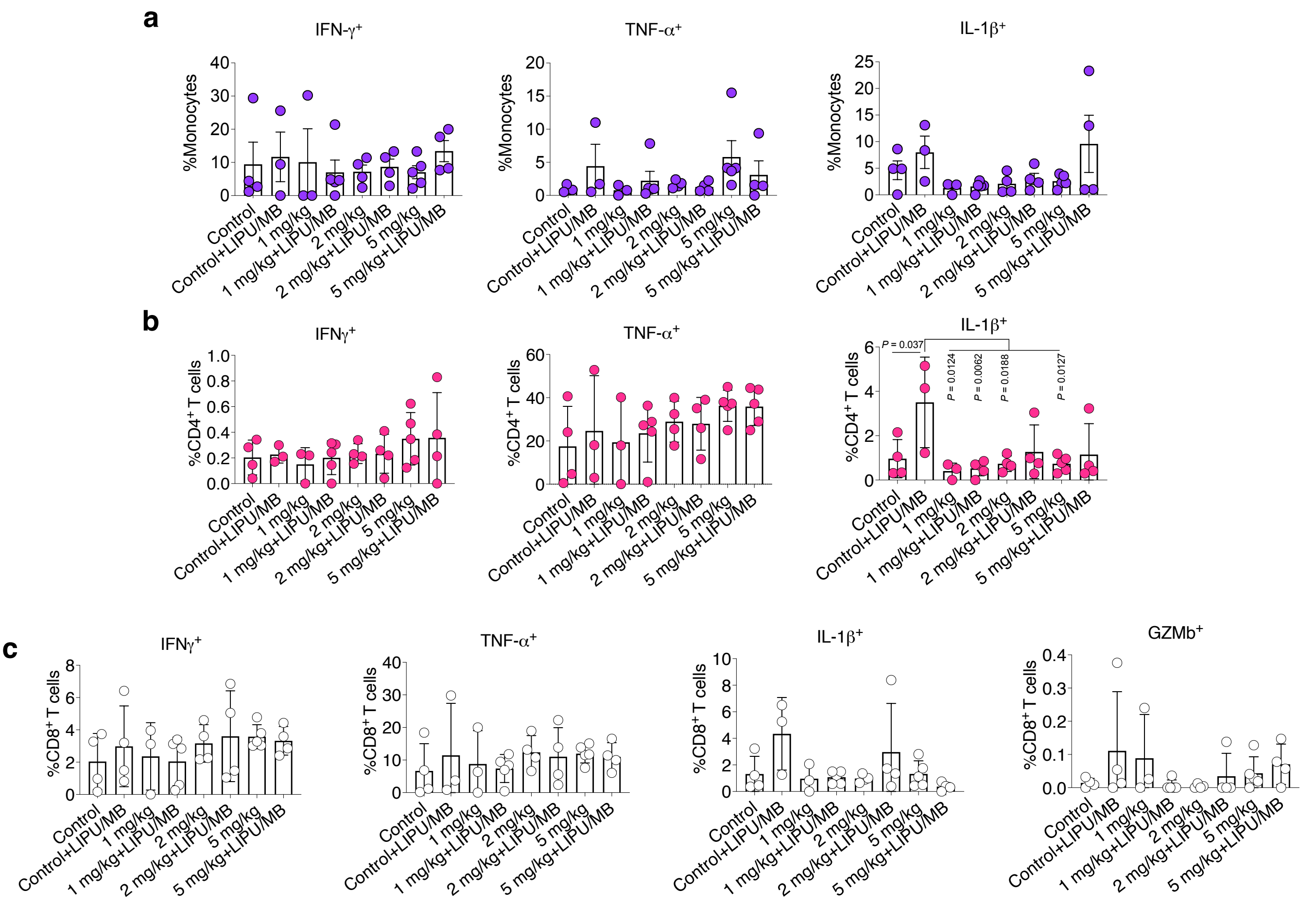

Supplementary Figure 6. Effect of liposomal DOX in peripheral immune cells. a, b, c, Bar plots showing the percentages of monocytes (**a**), $CD4^+$ (**b**), and $CD8^+$ (**c**) T cells producing IFN-γ^+, TNF-α^+, IL-1β^+, and GZMb from groups treated with different doses of liposomal DOX (1, 2, and 5 mg/kg) with or without LIPU/MB. n=3 mice for control+LIPU/MB and 1mg/kg; n=4 mice for control, 2 mg/kg, 2 mg/kg+LIPU/MB, and 5 mg/kg+LIPU/MB; and n=5 mice for 1 mg/kg+LIPU/MB and 5 mg/kg. All samples were derived from biologically independent mice from 1 experiment. *P* values were derived from one-way ANOVA with post hoc Tukey's multiple comparisons test. Source data are provided as a Source Data file. Data are presented as mean ± SEM.

Supplementary Figure 7

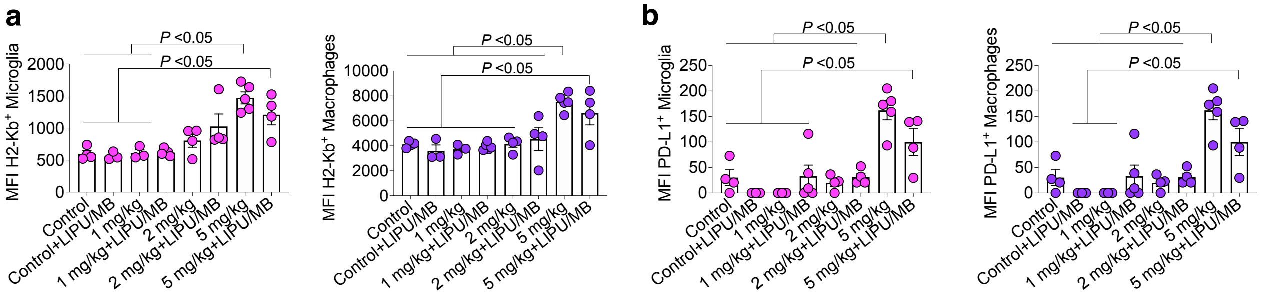

Supplementary Figure 7. Treatment with liposomal DOX induces the expression of H2-K^b and PD-L1 in GBM-infiltrating CD11b$^+$ myeloid cells. a, **b**, Bar plots showing the percentage of MHC I$^+$ (**a**) and PD-L1$^+$ (**b**) cells from microglia (left) and macrophages (right) from mouse groups treated with different doses of liposomal DOX. n=3 mice for control+LIPU/MB and 1mg/kg; n=4 mice for control, 2 mg/kg, 2 mg/kg+LIPU/MB, and 5 mg/kg+LIPU/MB; and n=5 mice for 1 mg/kg+LIPU/MB and 5 mg/kg. All samples were derived from biologically independent mice from 1 experiment. *P* values were derived from one-way ANOVA with post hoc Tukey's multiple comparisons test. Exact *P* values are provided in the Source Data file. Source data are provided as a Source Data file. Data are presented as mean ± SEM.

Supplementary Figure 8

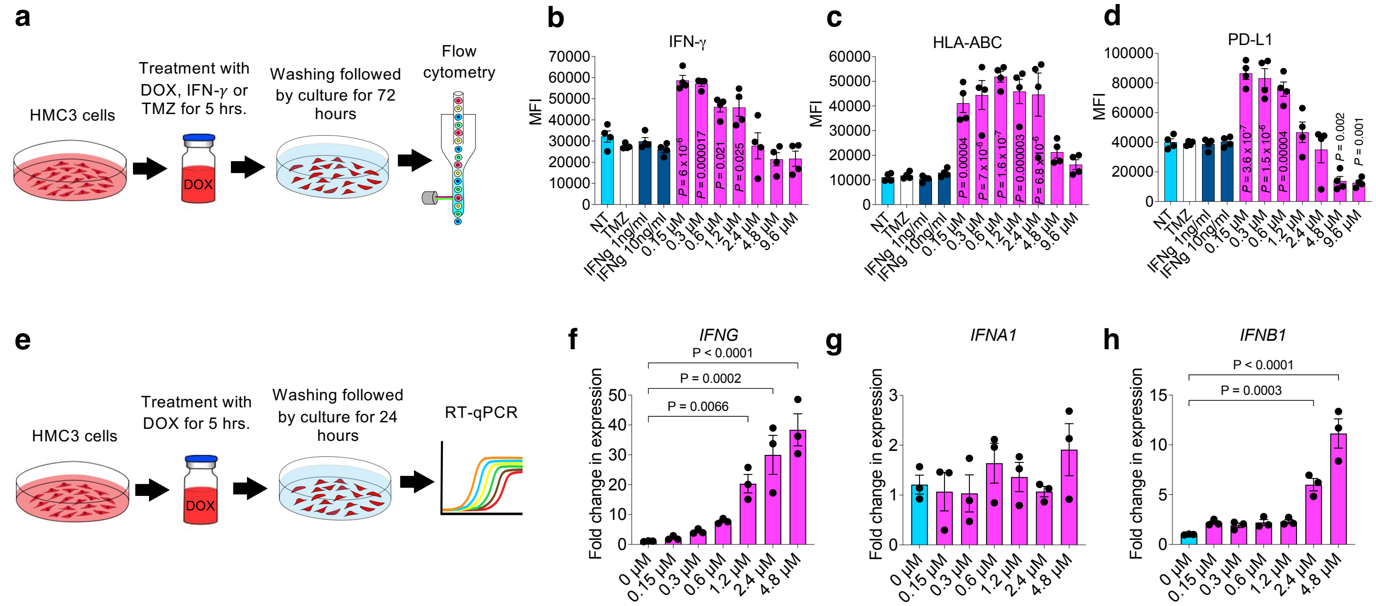

Supplementary Figure 8: DOX induces immunogenic responses in HMC3 cells. a, Schematic of the experimental workflow. HMC3 cells were treated with doxorubicin (DOX), interferon-gamma (IFN-γ), or temozolomide (TMZ) for 5 hours, followed by a washing step and subsequent culture for 72 hours. The cells were then analyzed by flow cytometry and RT-qPCR. **b**, MFI of IFN-γ expression in HMC3 cells post-treatment, showing dose-dependent increases with DOX and IFN-γ. **c**, MFI of HLA-ABC expression on HMC3 cells, indicating upregulation in response to DOX and IFN-γ treatments. **d**, MFI of PD-L1 expression on HMC3 cells, demonstrating increased levels following DOX and IFN-γ treatments, with a dose-dependent effect for DOX. n = 3 biological replicates per condition. **e**, Schematic illustrating the RT-qPCR workflow for analyzing cytokine gene expression post-DOX treatment. **f**, **g**, **h**, RT-qPCR results showing fold changes in gene expression of *IFNG* (**f**), *IFNA1* (**g**), and *IFNB1* (**h**) post-treatment with DOX. n = 3 biological replicates per condition. Statistical significance assessed by one-way ANOVA is indicated by asterisks: *P < 0.05, **P < 0.01, ***P < 0.001. Source data are provided as a Source Data file. Data are presented as mean ± SEM.

Supplementary Figure 9

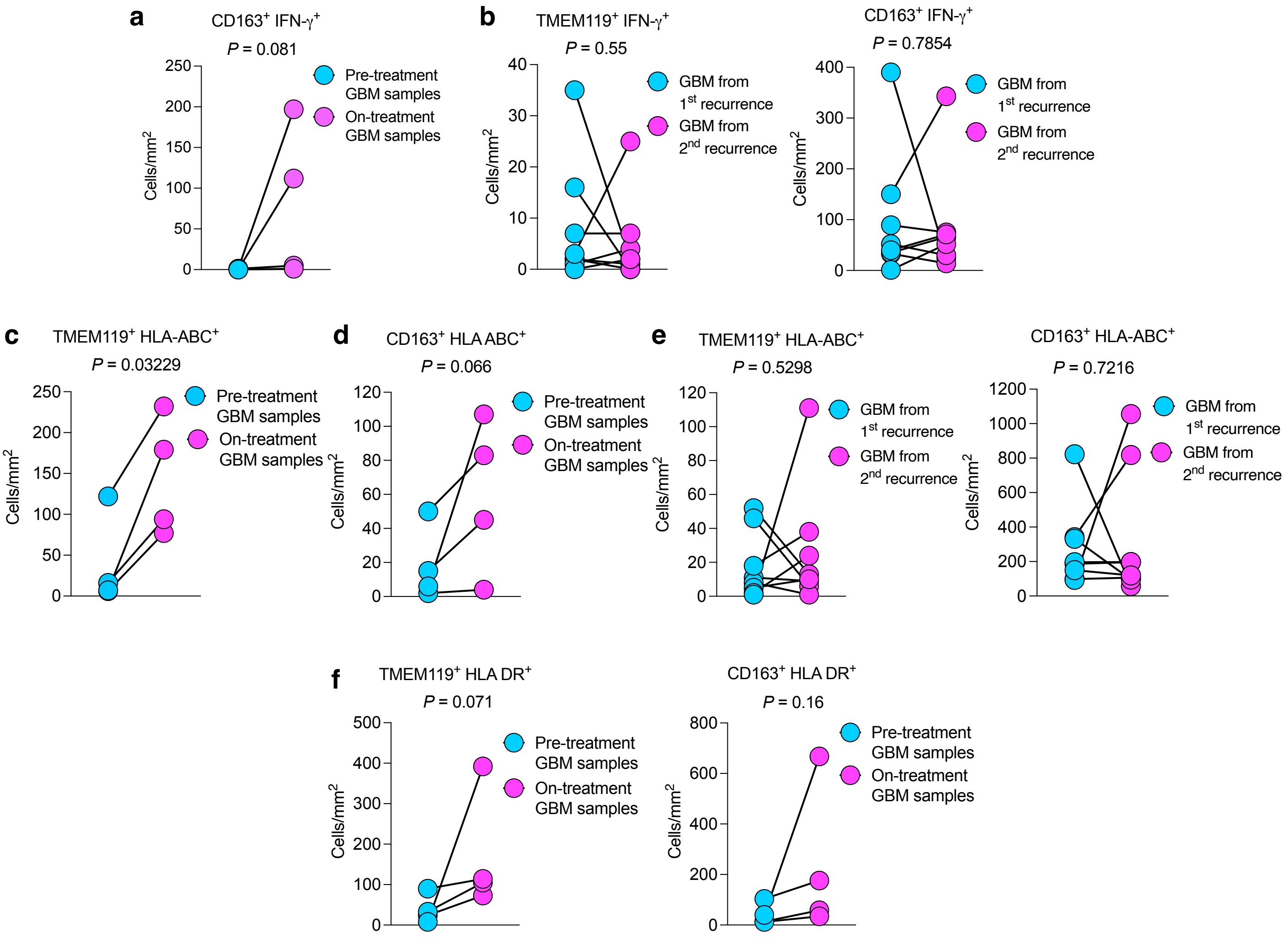

Supplementary Figure 9: Immunophenotypic changes in GBM samples before and after treatment. a, Dot plot displaying the density of IFN-γ expressing CD163+ myeloid cells in pre-treatment and on-treatment GBM samples. n = 4 paired GBM samples. **b**, Quantitative analysis of TMEM119$^+$ IFN-γ$^+$ (left) and CD163$^+$ IFN-γ$^+$ (right) cell densities in GBM samples from the first and second recurrences. **c**, **d**, Dot plot showing the cell density of TMEM119$^+$ HLA-ABC$^+$ (**c**) and CD163$^+$ HLA-ABC$^+$ (**d**) cells in pre-treatment GBM samples and on-treatment GBM samples. **e**, Analysis of TMEM119$^+$ HLA-ABC$^+$ (left) and CD163$^+$ HLA-ABC$^+$ (right) cell densities in GBM samples from first and second recurrences. Each point represents the cell density from an individual patient sample, with lines connecting samples from the same patient across different recurrences. n = 8 paired GBM samples in **b**, **c**, **d**, and **e**. **f**, Quantitative analysis of TMEM119$^+$ HLA-DR$^+$ (left) and CD163$^+$ HLA-DR$^+$ (right) cell densities in GBM samples from the first and second recurrences. n = 4 paired GBM samples. A mixed effects model was constructed considering DOX+aPD-1 treatment as a fixed effect and patients as a random effect influencing cell densities from the indicated phenotypes. *P* values were obtained by chi-squared tests of likelihood ratio tests of the full model with DOX+PD-1 treatment as a fixed effect against the model without the fixed effect.

Supplementary Figure 10

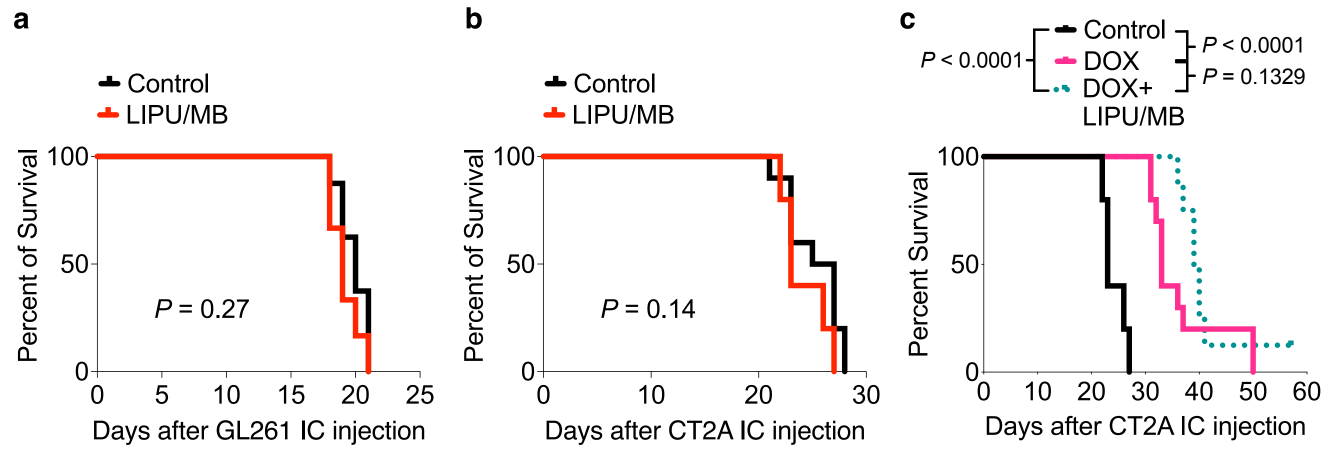

Supplementary Figure 10. Effect of LIPU/MB in toxicity and survival of murine GBM models. a, **b**, Kaplan-Meier curves of GBM-bearing mice undergoing and not undergoing sonication employing GL216 (**a**) and CT-2A (**b**) cells. GL261 survival study: n=8 mice for the control group, n=6 mice for the LIPU/MB group; CT-2A survival study: n=10 mice for the control group, n=10 mice for the LIPU/MB group. **c**, Kaplan-Meier curve showing survival of CT-2A-bearing mice treated with liposomal DOX with and without LIPU/MB (n=10 mice for the control group, n=10 mice for the DOX group, n=5 mice for the DOX+LIPU/MB group). *P* values derived from Log-rank test in **a**, **b**, and **c**.

Supplementary Figure 11

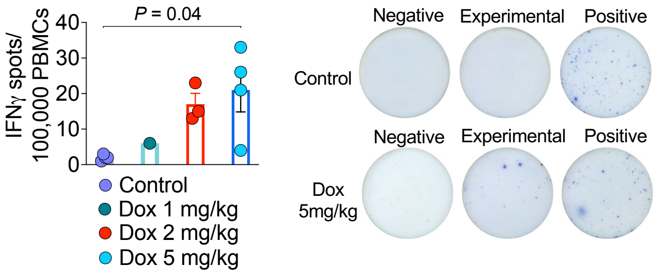

Supplementary Figure 11. Production of IFN-γ by PBMCs exposed to DOX. Bar plot showing the number of IFN-γ spots per 100,000 PBMCs extracted from GL261-bearing mice treated with the indicated doses of liposomal DOX. PBMCs from these animals were stimulated with GL261 cell lysate. Representative wells from the control and DOX 5 mg/kg are shown below the bar plots. n=3 mice for control group, n=1 mouse for the DOX 1 mg/kg group, n=3 mice for the DOX 2 mg/kg group, and n=4 mice for DOX 5 mg/kg. *P* values derived from one way-ANOVA with post hoc Tukey's multiple comparisons test. Source data are provided as a Source Data file. Data are presented as mean ± SEM.

Supplementary Figure 12

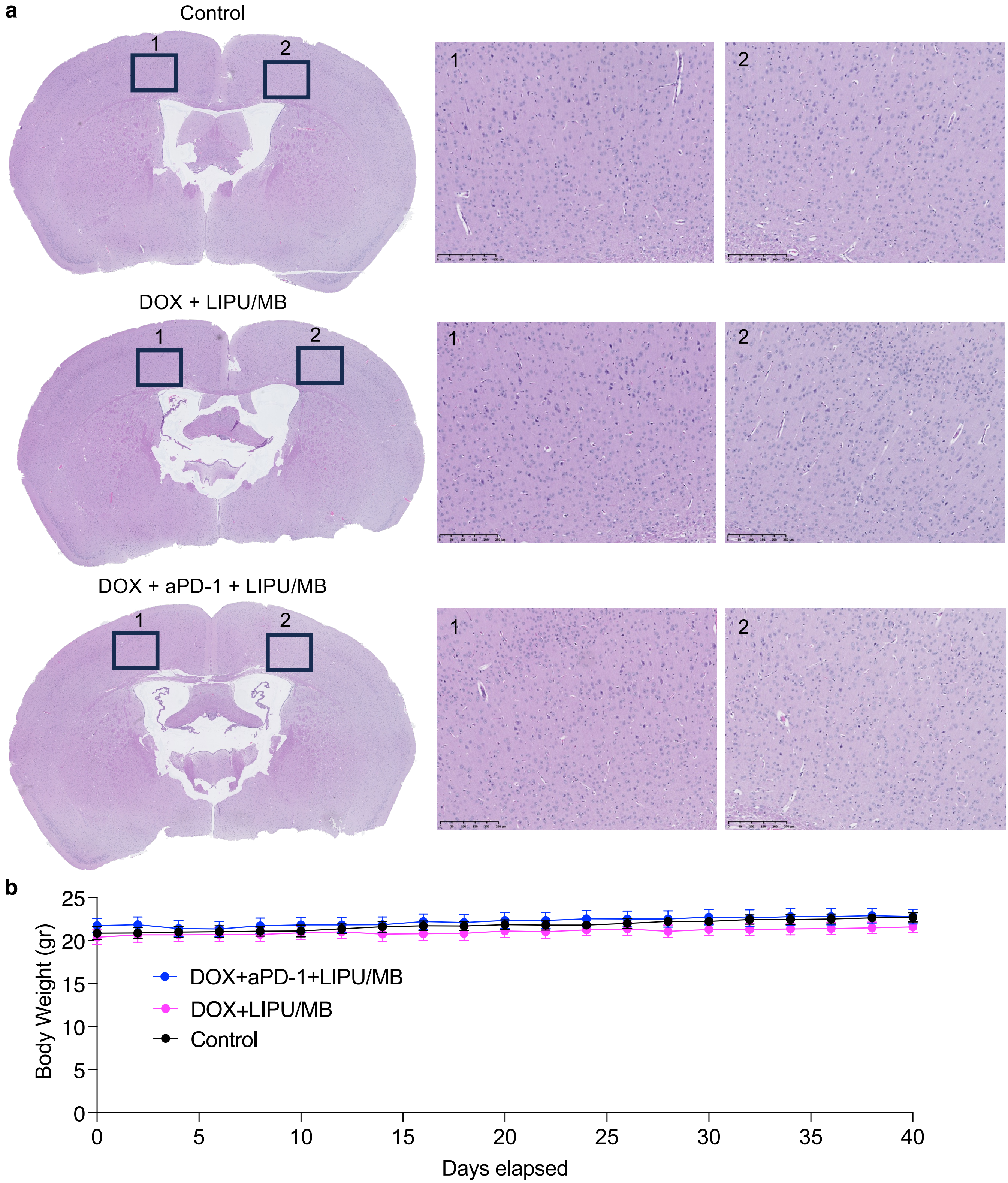

Supplementary Figure 12. Assessment of systemic and local toxicity in healthy mice following DOX plus aPD-1 delivered with LIPU/MB. a, H&E micrographs showing low (left) and high (right) magnification images of mouse brains from a control group that did not receive treatment, a DOX + LIPU/MB group, and from the group treated with DOX + aPD-1 + LIPU/MB. Images are representative of 3 mice per group. **b**, Line graph showing longitudinal monitoring of mouse body weight post-treatment with a therapeutic combination of DOX, aPD-1, and LIPU/MB. The control group (black line) and the treated group (blue line) are compared over 40 days. Body weight is a general indicator of health and potential systemic toxicity in murine models. The data suggests no significant weight loss across all groups, indicating that the treatment was well-tolerated without overt signs of systemic toxicity. n=5 mice for the control group, n=5 mice for 5 mg/kg DOX + LIPU/MB, and n=5 mice for 5 mg/kg DOX+aPD-1+LIPU/MB. Source data are provided as a Source Data file. Data are presented as mean ± SEM.

Supplementary Figure 13

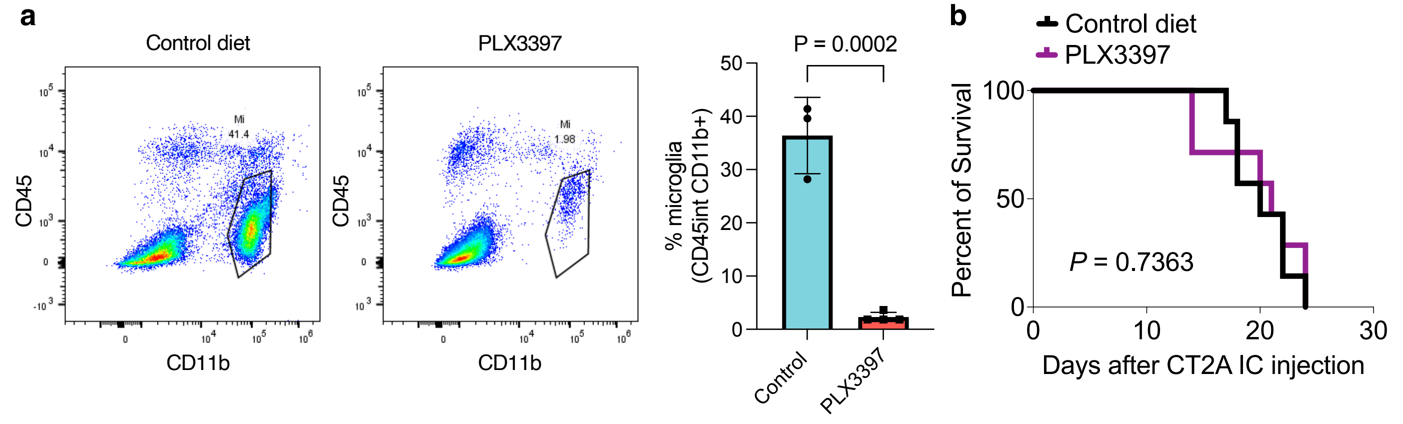

Supplementary Figure 13. Myeloid cells contribute to the efficacy of LIPU/MB in GBM-bearing mice. **a**, Representative flow cytometry plots (left) and bar plot (right) showing the depletion of brain myeloid cells. n=3 mice for the control diet group, n=4 mice for the PLX3397 group. *P* value derived from two-sided *t*-test. **b**, Kaplan-Meier curve showing the survival of CT-2A-bearing mice treated with either control diet or PLX3397 (n=7 per group). *P* value derived from Log-rank test. Source data are provided as a Source Data file. Data are presented as mean ± SEM in **a**.

Supplementary Figure 14

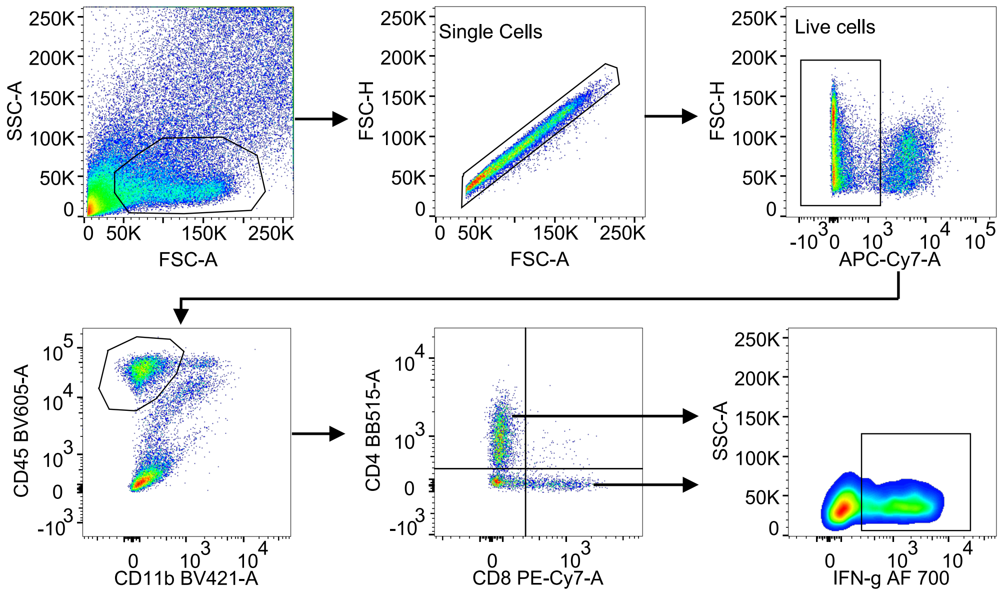

Supplementary Figure 14. Gating strategy used to analyze T cells from human GBMs treated and not treated with liposomal DOX plus aPD-1 delivered with LIPU/MB. Flow cytometry plots used to analyze the production of cytokines and the expression of surface markers on GBM-infiltrating T cells. Lymphocytes were gated based on SSC and FSC parameters followed by exclusion of doublets. Next, live cells were gated based on the viability staining and subsequently, lymphocytes were gated base on the expression of $CD45^+$ and $CD11b^-$. Then, lymphocytes were analyzed for expression of $CD8^+$ and $CD4^+$ markers. $CD8^+$ and $CD4^+$ T cells were evaluated for the expression of IFN-γ^+.

Supplementary Table 2. Primer list for qPCR

Gene	Organism	Primer	Primer sequences
IFNB1	Human	Fw	CGACACTGTTCGTGTTGTCA
	Human	Rv	GAAGCACAACAGGAGAGCAA
IFNA1	Human	Fw	GGAGAGGGTGGGAGAAACTC
	Human	Rv	AAGCGTGACCTGGTGTATGA
IFNG	Human	Fw	GTCCAACGCAAAGCAATACA
	Human	Rv	GCAGGCAGGACAACCATTAC
ACTB	Human	Fw	CATCCCCCAAAGTTCACAAT
	Human	Rv	ATGGCAAGGGACTTCCTGTA
