# Supplementary material for: Ultrasound-mediated delivery of doxorubicin to the brain results in immune modulation and improved responses to PD-1 blockade in gliomas
Source: Nat Commun. 2024 Jun 6;15:4698. doi: 10.1038/s41467-024-48326-w (PMC11156895; doi:10.1038/s41467-024-48326-w)
Supplement: Supplementary file 3 — Reporting Summary [file 41467_2024_48326_MOESM3_ESM.pdf]

nature portfolio

Corresponding author(s): Adam M. Sonabend

Last updated by author(s): Apr 12, 2024

Reporting Summary

Nature Portfolio wishes to improve the reproducibility of the work that we publish. This form provides structure for consistency and transparency in reporting. For further information on Nature Portfolio policies, see our Editorial Policies and the Editorial Policy Checklist.

Statistics

For all statistical analyses, confirm that the following items are present in the figure legend, table legend, main text, or Methods section.

n/a	Confirmed	
☐	☒	The exact sample size (n) for each experimental group/condition, given as a discrete number and unit of measurement
☐	☒	A statement on whether measurements were taken from distinct samples or whether the same sample was measured repeatedly
☐	☒	The statistical test(s) used AND whether they are one- or two-sided<br>*Only common tests should be described solely by name; describe more complex techniques in the Methods section.*
☐	☒	A description of all covariates tested
☐	☒	A description of any assumptions or corrections, such as tests of normality and adjustment for multiple comparisons
☐	☒	A full description of the statistical parameters including central tendency (e.g. means) or other basic estimates (e.g. regression coefficient) AND variation (e.g. standard deviation) or associated estimates of uncertainty (e.g. confidence intervals)
☐	☒	For null hypothesis testing, the test statistic (e.g. F, t, r) with confidence intervals, effect sizes, degrees of freedom and P value noted<br>*Give P values as exact values whenever suitable.*
☒	☐	For Bayesian analysis, information on the choice of priors and Markov chain Monte Carlo settings
☒	☐	For hierarchical and complex designs, identification of the appropriate level for tests and full reporting of outcomes
☒	☐	Estimates of effect sizes (e.g. Cohen's d, Pearson's r), indicating how they were calculated

Our web collection on statistics for biologists contains articles on many of the points above.

Software and code

Policy information about availability of computer code

Data collection	Vectra 3 Automated Quantitative Pathology Imaging System - AID EliSpot Reader - Cytation 5 multi-mode reader - BD FACSymphony Flow Cytometer- CFX Connect Real Time PCR system (BioRad) - 5500 Triple Quad equipped with an ExionLC™ AC20, SCIEX, Framingham, MA
Data analysis	inForm Tissue Finder version 2.6<br>Matlab version R2023b - tools: cyt3<br>FlowJo version 10.7.1 and 10.10<br>R version 4.3.0 - Packages: Phenoptr - PhenoptrReports - flowCore - shiny - reticulate - inForm2fcs - cytofkit2<br>R studio<br>GraphPad Prism version 10

For manuscripts utilizing custom algorithms or software that are central to the research but not yet described in published literature, software must be made available to editors and reviewers. We strongly encourage code deposition in a community repository (e.g. GitHub). See the Nature Portfolio guidelines for submitting code & software for further information.

Data

Policy information about availability of data

All manuscripts must include a data availability statement. This statement should provide the following information, where applicable:

- Accession codes, unique identifiers, or web links for publicly available datasets
- A description of any restrictions on data availability
- For clinical datasets or third party data, please ensure that the statement adheres to our policy

All data are available in the main text, supplementary materials, and Source Data files.

Research involving human participants, their data, or biological material

Policy information about studies with human participants or human data. See also policy information about sex, gender (identity/presentation), and sexual orientation and race, ethnicity and racism.

Reporting on sex and gender	The findings of this study do not apply to only one sex or gender. Source data reports sex data. Consent has been obtained from patients included in this study.
Reporting on race, ethnicity, or other socially relevant groupings	This study did not considered race, ethnicity, or other socially relevant groups as part of the hypothesis or analysis.
Population characteristics	Glioblastoma patients included in this research experienced a second tumoral recurrence. These patients were treated with standard of care (temozolomide plus radiotherapy) at initial tumor diagnosis. In addition, these patients were treated with albumin-bound paclitaxel delivered with ultrasound-mediated blood-brain barrier disruption when they experienced the first tumoral recurrence. Age and sex information is provided in the Supplementary File.
Recruitment	Recruited patients were part of a phase 1 clinical trial that evaluated skull-implantable ultrasound for the delivery of albumin-bound paclitaxel at the time of GBM progression (NCT04528680). After tumor progression following treatment in this trial, these patients consented and were enrolled in an expanded-access single-patient protocol where LIPU/MB was repurposed to deliver DOX and aPD-1 (pembrolizumab).
Ethics oversight	Institutional review board (IRB) approval was acquired from Northwestern University.

Note that full information on the approval of the study protocol must also be provided in the manuscript.

Field-specific reporting

Please select the one below that is the best fit for your research. If you are not sure, read the appropriate sections before making your selection.

[x] Life sciences [ ] Behavioural & social sciences [ ] Ecological, evolutionary & environmental sciences

For a reference copy of the document with all sections, see nature.com/documents/nr-reporting-summary-flat.pdf

Life sciences study design

All studies must disclose on these points even when the disclosure is negative.

Sample size	For experiments involving the analysis of human tissue samples, sample size was determined based on the number of all patients available for tumor analysis who were part of the expanded access protocols to receive treatment with doxorubicin and anti-PD-1 therapy. For in vitro real time PCR, survival studies, and flow cytometry experiments, sample size was determined based on pilot experiments that showed differences between groups. For all experiments, sample size is mentioned in the figure legends.
Data exclusions	No data was excluded from the analyses.
Replication	Experimental findings derived from mouse analyses were replicated in human analyses. The replicate numbers for each experiment are reported in the figure legends.
Randomization	For human studies, patients that progressed on the clinical trial NCT04528680 were started on Doxorubicin and anti-PD-1 delivered with LIPU/MB as part of expanded access protocols, and therefore randomization was not performed.<br>For animal studies, randomization was performed following intracranial tumor injection.
Blinding	Patients that progressed on Abraxane in the NCT04528680 trial were assigned to receive Doxorubicin and anti-PD-1 as salvage therapy and therefore blinding was not performed.<br>To validate our analysis, the regions of interest for the microscopy images used for multiplex immunofluorescence and H&E staining were delineated by a neuropathologist blinded to the treatment.

Reporting for specific materials, systems and methods

We require information from authors about some types of materials, experimental systems and methods used in many studies. Here, indicate whether each material, system or method listed is relevant to your study. If you are not sure if a list item applies to your research, read the appropriate section before selecting a response.

Materials & experimental systems

n/a	Involved in the study
☐	☒ Antibodies
☐	☒ Eukaryotic cell lines
☒	☐ Palaeontology and archaeology
☐	☒ Animals and other organisms
☒	☐ Clinical data
☒	☐ Dual use research of concern
☒	☐ Plants

Methods

n/a	Involved in the study
☒	☐ ChIP-seq
☐	☒ Flow cytometry
☐	☒ MRI-based neuroimaging

Antibodies

Antibodies used

For human flow cytometry experiments:
1. HLA-ABC AF-647 (dilution 1:100, Biolegend, 311414)
2. HLA-ABC FITC (dilution 1:100, Biolegend, 311404)
3. HLA-DR BV-421 (dilution 1:100, Biolegend, 307636)
4. IFN-γ AF-700 (dilution 1:100, Biolegend, 502520)
5. PD-L1 Pe-Cy7 (dilution 1:100, Biolegend, 374506)
7. CD45 BV605 (dilution 1:100, Biolegend, 368524)
8. CD3 PerCP (dilution 1:100, Biolegend, 300326)
9. CD8 PE-Cy7 (dilution 1:100, Biolegend, 300914)
10. CD4 FITC (dilution 1:100, Biolegend, 317408)

For mouse flow cytometry experiments:
1. CD8a BV605 (dilution 1:100, Biolegend, 100743)
2. CD4 FITC (dilution 1:100, Biolegend, 100510)
3. CD11b Pacific Blue (dilution 1:100, Biolegend, 101224
4. CD45 BV510 (dilution 1:100, Biolegend, 103138)
5. IFNg AF700 (dilution 1:100, Biolegend, 505824)
6. GZMb AF647 (dilution 1:100, Biolegend, 515406)
7. TNFa BV421 (dilution 1: 100, Biolegend, 502932)
8. IL-1B PE-Cy7 (dilution 1:100, nvitrogen, 25-7114-82)
9. H2-Kb BV421 (dilution 1:100, Biolegend, 116514)
10. PD-L1 PE-Cy7 (dilution 1:100, Biolegend, 124314)

For the multiplex immunofluorescence experiments:
1. TMEM119 (cat. HPA051870, Sigma-Aldrich, dilution 1:250, pH6) paired with Opal 520
2. CD163 (cat. ab182422, Abcam, clone EPR19518, dilution 1:600, pH9) paired with Opal 620
3. IFN-γ (cat. ab231036, Abcam, clone IFNG/466, dilution 1 ug/mL, pH9) paired with Opal 570
4. HLA-DR (cat, ab20181, Abcam, clone TAL 1B5, dilution 1:1000, pH6) paired with Opal 650
5. HLA-ABC (cat. ab70328, Abcam, clone EMR8-5, dilution 0.3 ug/mL, pH6) paired with Opal 690
6. SOX2 (cat. ab92494, Abcam, clone EPR3131, dilution 1:5000, pH9) paired with Opal 540

Validation

Antibodies for flow cytometry used in this study are commercially available and validated by the manufacturers and have cited in several publications.

For human flow cytometry experiments:
1. HLA-ABC AF-647: https://www.biolegend.com/de-at/products/alexa-fluor-647-anti-human-hla-a-b-c-antibody-2900?GroupID=BLG5954
2. HLA-ABC FITC: https://www.biolegend.com/de-at/products/fitc-anti-human-hla-a-b-c-antibody-1871
3. HLA-DR BV-421: https://www.biolegend.com/de-at/products/brilliant-violet-421-anti-human-hla-dr-antibody-7226
4. IFN-γ AF-700: https://www.biolegend.com/de-at/products/alexa-fluor-700-anti-human-ifn-gamma-antibody-3440
5. PD-L1 Pe-Cy7: https://www.biolegend.com/de-at/products/pe-cyanine7-anti-human-cd274-b7-h1-pd-l1-antibody-15790
7. CD45 BV605: https://www.biolegend.com/de-at/products/brilliant-violet-605-anti-human-cd45-antibody-14717
8. CD3 PerCP: https://www.biolegend.com/de-at/products/percp-anti-human-cd3-antibody-5612
9. CD8 PE-Cy7: https://www.biolegend.com/de-at/products/pe-cyanine7-anti-human-cd8a-antibody-1916
10. CD4 FITC: https://www.biolegend.com/de-at/products/fitc-anti-human-cd4-antibody-3653

For mouse flow cytometry experiments:
1. CD8a BV605: https://www.biolegend.com/de-at/products/brilliant-violet-605-anti-mouse-cd8a-antibody-7636
2. CD4 FITC: https://www.biolegend.com/de-at/products/fitc-anti-mouse-cd4-antibody-480
3. CD11b Pacific Blue: https://www.biolegend.com/de-at/products/pacific-blue-anti-mouse-human-cd11b-antibody-3863
4. CD45 BV510: https://www.biolegend.com/de-at/products/brilliant-violet-510-anti-mouse-cd45-antibody-7995
5. IFNg AF700: https://www.biolegend.com/de-at/products/alexa-fluor-700-anti-mouse-ifn-gamma-antibody-12538
6. GZMb AF647: https://www.biolegend.com/de-at/products/alexa-fluor-647-anti-human-mouse-granzyme-b-antibody-6067
7. TNFa BV421: https://www.biolegend.com/de-at/products/brilliant-violet-421-anti-human-tnf-alpha-antibody-7215
8. IL-1B PE-Cy7: https://www.thermofisher.com/antibody/product/IL-1-beta-Pro-form-Antibody-clone-NJTEN3-Monoclonal/25-7114-82
9. H2-Kb BV421: https://www.biolegend.com/de-at/products/pacific-blue-anti-mouse-h-2kb-antibody-6298
10. PD-L1 PE-Cy7: https://www.biolegend.com/de-at/products/pe-cyanine7-anti-mouse-cd274-b7-h1-pd-l1-antibody-6721

For the multiplex immunofluorescence experiments:
1. TMEM119: https://www.sigmaaldrich.com/US/en/product/sigma/hpa051870
2. CD163: https://www.abcam.com/products/primary-antibodies/cd163-antibody-epr19518-ab182422.html
3. IFN-γ: https://www.abcam.com/products/primary-antibodies/interferon-gamma-antibody-epr21704-ab231036.html
4. HLA-DR: https://www.abcam.com/products/primary-antibodies/hla-dr-antibody-tal-1b5-ab20181.html
5. HLA-ABC: https://www.abcam.com/products/primary-antibodies/hla-class-1-abc-antibody-emr8-5-ab70328.html
6. SOX2: https://www.abcam.com/products/primary-antibodies/sox2-antibody-epr3131-ab92494.html

Eukaryotic cell lines

Policy information about cell lines and Sex and Gender in Research

Cell line source(s)	The murine glioma GL261 cell line was acquired from the National Institutes of Health<br>The murine glioma CT2A cell line was acquired from Millipore<br>HMC3 cells were acquired from ATCC<br>The patient-derived xenografts (PDXs) GBM6 and GBM63 were provided by Dr. Jann Sarkaria from Mayo Clinic.
Authentication	All cell lines used were authenticated by short tandem repeat (STR) analysis
Mycoplasma contamination	Human cell lines tested negative for mycoplsma contamination. All murine cell lines used for this study were routinely tested for mycoplasma and were confirmed negative before intracranial orthotopic injection
Commonly misidentified lines (See ICLAC register)	The cell lines used are not registered as commonly misidentified lines by ICLAC

Animals and other research organisms

Policy information about studies involving animals; ARRIVE guidelines recommended for reporting animal research, and Sex and Gender in Research

Laboratory animals	All animal experiments were approved by Northwestern University's Institutional Animal Care and Usage Committee under protocol no. IS000017464. Six- to twelve-week-old male and female C57BL/6 mice were purchased from Charles River Laboratories for these experiments. Sex was not considered in the study design because this variable was not relevant to the study. All animals were housed in a pathogen-free animal facility at Northwestern University at a relatively constant temperature of 24°C and humidity of 30%–50%. The Cd8-/- mice breeders were purchased from the Jackson Laboratory (B6.129S2-Cd8atm1Mak/J, stock #002665). The genotyping protocol was performed following the recommendation of the Jackson Laboratory and separated by gel electrophoresis on a 1.5% agarose gel. Equal ratio of male and female mice were used for all the experiments.
Wild animals	The study did not involve wild animals
Reporting on sex	Sex was not considered as an important variable in the study design.
Field-collected samples	The study did not involve samples collected from the field
Ethics oversight	Studies involving animals were approved by Northwestern University's Institutional Animal Care and Usage Committee under the protocol no. IS000017464

Note that full information on the approval of the study protocol must also be provided in the manuscript.

Plants

Seed stocks | NA

Novel plant genotypes | NA

Authentication | NA

Flow Cytometry

Plots

Confirm that:

- [x] The axis labels state the marker and fluorochrome used (e.g. CD4-FITC).
- [x] The axis scales are clearly visible. Include numbers along axes only for bottom left plot of group (a 'group' is an analysis of identical markers).
- [x] All plots are contour plots with outliers or pseudocolor plots.
- [x] A numerical value for number of cells or percentage (with statistics) is provided.

Methodology

Sample preparation

Immunophenotyping and treatment of GBM-bearing mice with increasing doses of liposomal DOX. Mice were bled retro-orbitally, and blood samples were collected in heparinized PBS solution (1 mg/mL, Sigma-Aldrich). Red blood cells were lysed using an ACK lysing solution (Gibco, Thermo Fisher). After blood collection, mice were euthanized in a CO2 chamber and intracardially perfused with chilled PBS. PBMCs were isolated from whole blood using Ficoll-Paque density gradient centrifugation. Whole blood was diluted 1:1 with sterile phosphate-buffered saline (PBS). The diluted blood was carefully layered over an equal volume of Ficoll-Paque in a centrifuge tube, ensuring a clear separation of layers. The samples were then centrifuged at 400 g for 30 minutes at room temperature, with the centrifuge brake deactivated to allow undisturbed layer separation. Post-centrifugation, the sample separated into four distinct layers. The thin, white PBMC layer located above the Ficoll-Paque was carefully aspirated using a pipette and transferred into a new centrifuge tube. The cells were washed by adding PBS and centrifuging at 200-300 g for 10 minutes. The supernatant was discarded, and the cell pellet was resuspended in fresh PBS. This washing step was repeated to ensure the complete removal of any residual Ficoll-Paque and platelets. The final cell pellet was resuspended in an appropriate medium for subsequent viability assessment using the trypan blue exclusion method. The isolated PBMCs were then used immediately for antibody staining.
Brain single-cell suspensions were obtained by mechanical dissociation using a manual tissue homogenizer (Potter-Elvehjem PTFE pestle, Sigma-Aldrich) in HBSS. Myelin and debris were removed by Percoll gradient separation. Brain single-cell suspensions were filtered through a 70-mm cell strainer and a syringe plunger. Cells were washed with complete RPMI media and were used immediately for antibody staining.

Flow cytometry and immunophenotype analysis. Immunophenotype analysis of brain single-cell suspension and PBMCs was performed from different DOX treatment groups. After collection single-cell suspensions, cells were counted and washed with staining buffer (5% bovine serum albumin, 0.001% sodium azide in PBS). Next, cells were incubated for 5 minutes with human Fc block (BD 564219) on ice and were stained for surface markers for 30 min at 4C with the following fluorescently conjugated antibodies: CD45 BV605 (cat. 368524, Biolegend), CD3 PerCP (cat. 300326, Biolegend), CD8 PE-Cy7 (cat. 300914, Biolegend), CD4 FITC (cat. 317408, Biolegend), H2-Kb (cat. 116514, Biolegend), and PD-L1 (cat. 124314, Biolegend). Cells were washed twice with cold PBS. Cells were stained with Fixable Viability Dye eFluor 780 (eBioscience, Thermo Fisher) for 30 minutes at 4C. Cells were washed twice with the staining buffer. For the detection of cytokine production, cells were fixed and permeabilized using the eBioscience Foxp3/Transcription Factor Staining Buffer Set (Invitrogen, Thermo Fisher) for 90 minutes at room temperature. Cells were washed twice with the Permeabilization Buffer (provided in the permeabilization/fixation buffer kit) and incubated with 1 mL Ab for 1 hour at 4C. Cells were washed twice with the Permeabilization Buffer (provided in the permeabilization/fixation buffer kit) and incubated with 1 mL Ab for 1 hour at 4C. Cells were washed twice with staining buffer. To evaluate cytokine expression, cells were stimulated for 5 hours at 37C with the eBioscience Cell Stimulation Cocktail plus protein transport inhibitors (500x, Thermo Fisher) prior staining. Cells were washed twice with the staining buffer and stained with antibodies against IFN-γ (cat. 505824, Biolegend), GZMb (cat. 515406, Biolegend), TNF-α (cat. 502932, Biolegend), and IL-1β (cat. 25-7114-82, Invitrogen).

Processing and flow cytometry analysis of human GBM-infiltrating T cells. Non-treated and DOX-treated GBM samples were acquired by the Nervous System Tumor Bank at Northwestern University. Tumor samples were immediately processed into single-cell suspension using the Adult Brain Dissociation Kit (cat. 130-107-677, Miltenyi Biotec) following the manufacturer's protocol. Single-cell suspension of GBM samples was cryopreserved using RPMI media (Corning), DMSO, and FBS (Hyclone). Cryopreserved non-treated and DOX-treated single-cell suspensions were thawed at the same time for stimulation and staining. Cells were washed with complete RPMI media and re-stimulated with a cell activation cocktail (cat. 423303, Biolegend) for 4 hours. After 4 hours of re-stimulation, cells were washed with 1X PBS and stained with Zombie-NIR (cat.

	423105, Biolegend) for cell viability. Next, cells were incubated for 5 minutes with human Fc block (BD 564219) on ice and were stained for surface markers with the following fluorescently conjugated antibodies: CD45 BV605 (cat. 368524, Biolegend), CD3 PerCP (cat. 300326, Biolegend), CD8 PE-Cy7 (cat. 300914, Biolegend), CD4 FITC (cat. 317408, Biolegend). Cells were then fixed with Fixation/Permeabilization concentrate (cat. 00-5123-43, eBiosciences) and stained intracellularly with IFN-γ Alexa Fluor 700 (cat. 505824, Biolegend). Data was acquired using BD FACSymphony Flow Cytometer and analyzed using FlowJo (BD).
Instrument	Data were acquired on BD FACSymphony Flow Cytometer
Software	All the analyses were conducted using FlowJo version 10.7.1 and 10.10, Matlab version R2023b - tools: cyt3, and R version 4.3.0
Cell population abundance	All cells from single-cell suspensions derived from brain, spleen, and blood were analyzed. No sorting was performed in these experiments.
Gating strategy	For murine glioma immunophenotyping we used an SSC-A vs FCS-A density plot. Then, we gated immune cells based on their known location within these parameters. Then, cells were gated for singlets using an FSC-H vs FSC-A plot. The lymphocyte/ singlet gate was further analyzed to identify live/dead cells. After selecting for live cells, CD45+ vs CD11b plot was generated. Microglia were identified as CD11b positive CD45 negative cells, while macrophages were identified as CD11b - CD45 positive cells . Finally, CD45 positive - CD11b negative cells were analyzed for CD4 and CD8 staining.<br><br>For profiling of GBM-infiltrating lymphocytes, lymphocytes were gated based on SSC and FSC parameters followed by exclusion of doublets. Next, live cells were gated based on the viability staining and subsequently, lymphocytes were gated base on the expression of CD45+ and CD11b-. Then, lymphocytes were analyzed for expression of CD8+ and CD4+ markers. CD8+ and CD4+ T cells were evaluated for the expression of IFN-g+.

☒ Tick this box to confirm that a figure exemplifying the gating strategy is provided in the Supplementary Information.

Magnetic resonance imaging

Experimental design

Design type	The MRIs obtained were standard T1 with and without contrast MRIs used for neuronavigation. No functional MRIs were taken.
Design specifications	N/A
Behavioral performance measures	N/A

Acquisition

Imaging type(s)	Structural, Diffusion, Perfusion
Field strength	3T
Sequence & imaging parameters	Siemens 3T<br>T1w spin echo (3D, 256x256, 120 flip angle, 0.9375mm x 0.9375 mm, 0.9mm slice thickness)<br>FLAIR (2D, 256x240, 0.43 mm x 0.43 mm, 3 mm slice thickness)<br>Diffusion (2D, 284 x 384, 0.6 mm x 0.6 mm, 3 mm slice thickness)
Area of acquisition	Whole brain scan

Diffusion MRI ☒ Used ☐ Not used

Parameters *Specify # of directions, b-values, whether single shell or multi-shell, and if cardiac gating was used.*

Preprocessing

Preprocessing software	MRI were not used for analysis
Normalization	N/A
Normalization template	N/A
Noise and artifact removal	N/A
Volume censoring	N/A

Statistical modeling & inference

Model type and settings	N/A

Effect(s) tested	N/A

Specify type of analysis: ☐ Whole brain ☐ ROI-based ☐ Both

Statistic type for inference (See Eklund et al. 2016)	N/A
Correction	N/A

Models & analysis

n/a	Involved in the study
☒	☐ Functional and/or effective connectivity
☒	☐ Graph analysis
☒	☐ Multivariate modeling or predictive analysis
